# Assessment of Harmful Emissions from Multiple Binder Systems in Pilot-Scale Sand Casting

**DOI:** 10.3390/molecules30132765

**Published:** 2025-06-27

**Authors:** Erika Garitaonandia, Andoni Ibarra, Angelika Kmita, Rafał Dańko, Mariusz Holtzer

**Affiliations:** 1 AZTERLAN, Basque Research and Technology Alliance (BRTA), Aliendalde Auzunea, n°6, 48200 Durango, Spain; 2 Academic Centre for Materials and Nanotechnology, AGH University of Krakow, A. Mickiewicza 30 St., 30-059 Krakow, Poland; akmita@agh.edu.pl; 3 Faculty of Foundry Engineering, AGH University of Krakow, A. Mickiewicza 30 St., 30-059 Krakow, Poland; rd@agh.edu.pl

**Keywords:** foundry emissions, hazardous pollutants, inorganic binders, occupational health, volatile organic compounds, polycyclic aromatic hydrocarbons

## Abstract

This study investigates hazardous emissions from foundry binder systems, comparing organic resins (phenolic urethane, furan, and alkaline-phenolic) and clay-bonded green sand with inorganic alternatives (sodium silicate and geopolymer). The research was conducted at the Fundaciόn Azterlan pilot plant (Spain), involving controlled chamber tests for the production of 60 kg iron alloy castings in 110 kg sand molds. The molds were evaluated under two configurations: homogeneous systems, where both mold and cores were manufactured using the same binder (five trials), and heterogeneous systems, where different binders were used for mold and cores (four trials). Each mold was placed in a metallic box fitted with a lid and an integrated gas extraction duct. The lid remained open during pouring and was closed immediately afterward to enable efficient evacuation of casting gases through the extraction system. Although the box was not completely airtight, it was designed to direct most exhaust gases through the duct. Along the extraction system line, different sampling instruments were strategically located for the precise measurement of contaminants: volatile organic compounds (VOCs), polycyclic aromatic hydrocarbons (PAHs), phenol, multiple forms of particulate matter (including crystalline silica content), and gases produced during pyrolysis. Across the nine trials, inorganic binders demonstrated significant reductions in gas emissions and priority pollutants, achieving decreases of over 90% in BTEX compounds (benzene, toluene, ethylbenzene, and xylene) and over 94% in PAHs compared to organic systems. Gas emissions were also substantially reduced, with CO emissions lowered by over 30%, NO_x_ by more than 98%, and SO_2_ by over 75%. Conducted under the Greencasting LIFE project (LIFE 21 ENV/FI/101074439), this work provides empirical evidence supporting sodium silicate and geopolymer binders as viable, sustainable solutions for minimizing occupational and ecological risks in metal casting processes.

## 1. Introduction

Iron casting is a critical process in the manufacturing sector, playing a vital role in the production of components for the automotive, shipbuilding, construction, and mechanical engineering industries. The foundry industry—particularly in Europe, as the world’s second-largest producer (16.8 million tons, including light metals) of castings—faces increasing challenges in balancing production efficiency, product quality, and environmental sustainability [1]. Sand casting remains the predominant method for manufacturing metal components, with the binders used in the production of molds and cores having some of the most significant environmental impacts in this context.

Organic binders, including various forms of resins and hardeners, have long dominated sand casting technology alongside bentonite-based green sand [2]. However, the past decade has seen a renewed interest in inorganic binders, driven by stricter environmental demands and the need for improved casting performance.

This resurgence of inorganic binders began primarily in the aluminum casting sector, where modified silicate—based binders have been successfully implemented in the production of critical automotive components, such as engine heads and blocks [3]. Their success in aluminum foundries has sparked growing interest in transferring these benefits to iron and steel casting operations [4]. As of 2020, only 1% of European foundries used inorganic systems for mold and core manufacturing, with most of these being light metal foundries. In Germany, for instance, 11 out of 297 foundry companies use inorganic binders, primarily in the aluminum sector [5].

One of the key drivers behind the renewed focus on inorganic binders is their significantly lower environmental impact, since water is used as a solvent. Organic binders, while effective, are associated with substantial emissions of volatile organic compounds (VOCs), including hazardous substances such as BTEX compounds (benzene, toluene, ethylbenzene, and xylene) and polycyclic aromatic hydrocarbons (PAHs) [6,7,8,9,10,11].

Previous laboratory-scale studies conducted as part of the Greencasting LIFE project have reported significant emission reductions when comparing various inorganic binders to organic phenolic urethane systems [12,13]. These studies demonstrated over 98% reductions in BTEX emissions and more than 94% for PAHs. However, despite these promising results, a significant research gap remains: few studies have been conducted under real foundry conditions. Specifically, there is a lack of analyses using quantities of sand and metal that are more representative of industrial scales, as well as considering different combinations of binders in molds and cores.

The current investigation evaluates both inorganic and organic binders under real foundry conditions, analyzing conventional no-bake chemical systems such as furan, alkaline phenolic, and phenolic urethane, alongside green sand molds. For inorganic options, it specifically examines geopolymers and sodium silicate-based systems. The study explores diverse combinations of mold/core materials to assess binder interactions and emission patterns throughout the iron casting process.

The study employs a controlled pouring methodology, in which molten iron is cast into molds within a dedicated testing chamber optimized for controlled exhaust gas capture. This experimental design systematically reduces environmental interference while enabling direct performance comparisons between molding systems. By precisely tracking the pyrolysis and combustion byproducts generated during the thermal decomposition of binders, this study offers valuable insights into the environmental impacts of various foundry practices, contributing to a more comprehensive understanding of sustainable casting processes.

These results will be crucial for guiding the practical implementation of cleaner technologies in the foundry industry, potentially influencing future regulations and industrial practices.

Despite these advancements, challenges remain in the industrial adoption of inorganic binder technology for iron casting. Foundries must navigate the technical hurdles associated with integrating new materials into existing production workflows, with each facility likely requiring specific adjustments tailored to its unique operational characteristics.

## 2. Results and Discussion

In the following subsections, the emission results from the nine test molds are presented. Molds were evaluated under two configurations: homogeneous systems, where both mold and cores were manufactured using the same binder (five trials), and heterogeneous systems, where mold and cores used different binders (four trials).

Table 1 lists the test codes for each mold/core designation.

These combinations were carefully selected based on both pH levels and the compatibility of materials used in molds and cores. This approach was adopted with the specific goal of enabling future implementation in foundry operations.

For a detailed description of the mold and binder compositions, see Section 3 (Materials and Methods).

### 2.1. BTEX Emissions

**Figure 1:**
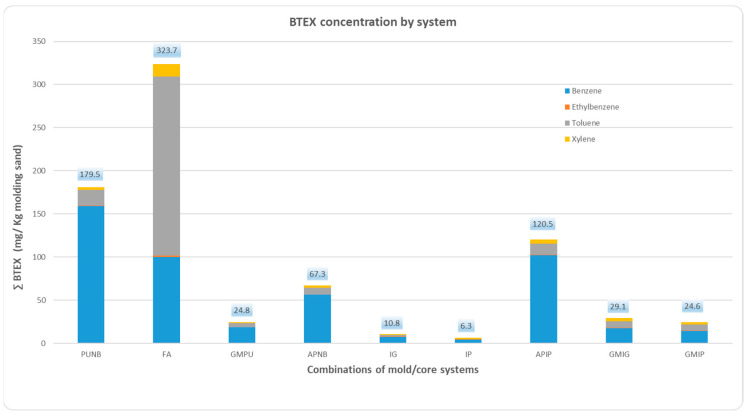
BTEX emissions of each system and combinations and individual BTEX compounds, calculated per kilogram of molding sands (specific numerical values provided in Table 2).

Quantitative analysis of benzene, toluene, ethylbenzene, and xylene emissions, expressed in mg/kg of sand mixture, revealed significant variations across binder systems, as depicted in Figure 1 and Table 2. The data reflect BTEX emissions for each binder system during the first 60 min after casting. In Figure 1, each bar represents one of the nine tested mold/core combinations, with the concentrations of individual BTEX compounds grouped together. The results show that the total BTEX emissions were highest for FA, followed by PUNB and APNB. Green sand emissions fell within an intermediate range. Notably, inorganic binder systems produced the lowest BTEX emissions, achieving reductions of 97% relative to the FA system, 95% relative to PUNB, 90% relative to the alkaline phenolic system, and over 65% compared to green sand molding systems.

The distribution of BTEX compounds varied markedly among the nine systems analyzed. Benzene was the predominant compound in most systems, with particularly high levels observed in the organic systems PUNB, APNB, and APIP, where it accounted for over 83% of total BTEX emissions. In contrast, the FA system exhibited a lower proportion of benzene (31%), with toluene being the dominant compound at 64%. Toluene also presented the highest relative abundance in the FA system (64%), followed by green sand molding systems (>20%). Ethylbenzene levels were nearly undetectable in all cases.

No significant differences were observed between the mold/core combinations made with GMPU core and both GMIG and GMIP.

### 2.2. PAH Emissions

**Figure 2:**
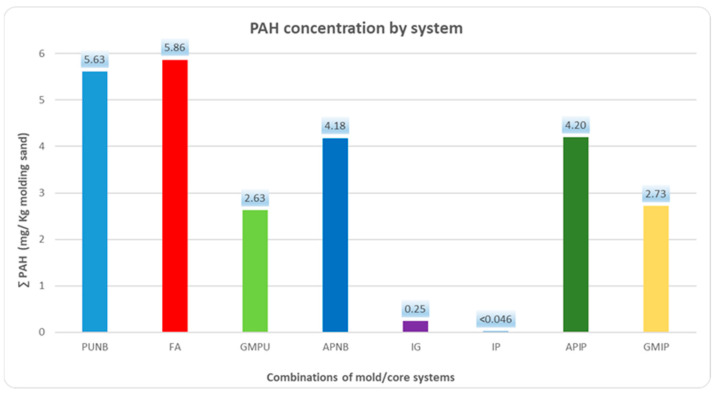
Total PAH emissions for each system, calculated per kilogram of molding sands.

The emission profile for polycyclic aromatic hydrocarbons (PAHs) followed a similar pattern to BTEX, as shown in Figure 2 and Table 2. The data reflect the PAH emissions for each binder system during the first 60 min after casting. The results show that FA produced the highest PAH emissions, followed by PUNB, APIP and APNB. Green sand systems remained at an intermediate level. Inorganic binders (IG and IP) achieved reductions of over 94% compared to organic binders and over 90% compared to green molding sands.

Each system is characterized by the predominance of one or two specific PAHs, with naphthalene and acenaphthene + fluorene being the most recurrent major compounds. (Table 2).

No significant differences were observed among several mold/core combinations. Specifically, the emissions were similar between green sand molds paired with either PUNB cores or inorganic cores.

### 2.3. Phenol Emissions

Phenol emissions were found to be below the limit of detection of the analysis laboratory for all tested binder systems (Table 2). Decarboxylation and dehydroxylation reactions were considered to have converted phenol into benzene.

### 2.4. Crystalline Silica and Particulate Matter

Crystalline silica levels were below detection limits for all systems except FA. Particulate matter emissions were highest in FA and PUNB, as shown in Table 2. GMPU also showed notable particulate emissions, while the IP system exhibited the lowest particulate matter emissions among all tested systems.

The variation in particulate emissions is influenced by the pouring method, with the velocity and the temperature at which the liquid metal enters the mold significantly affecting the number of particles emitted. Higher pouring speeds and temperatures tend to generate more particulate matter, while slower, controlled pouring can reduce emissions. This relationship between pouring technique and particulate emissions highlights the importance of optimizing the casting process to minimize environmental impact.

### 2.5. Emission of CO, NO_x_, SO_2_, O_2_ and Temperature Profiles

The results of gas emissions for CO, O_2_, NO_x_, and SO_2_ during the pouring process reveal significant differences among the tested molding sands. The data presented in Table 2 show the maximum emission values for these gases across the nine tested systems.

#### 2.5.1. CO Emission Profile Analysis

**Figure 3:**
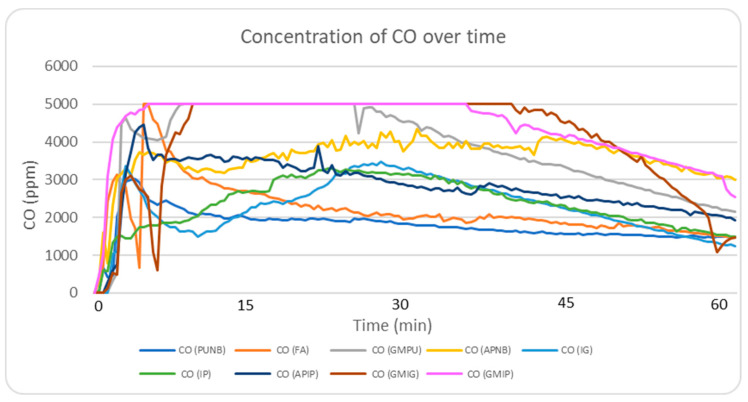
Real-time monitoring of CO concentrations during the experimental period for the nine different systems (note that 5000 ppm is the upper limit of the equipment).

The highest CO emission peaks were observed in green sand systems (>5000 ppm), followed by organic binder systems, mainly furan and alkaline phenolic systems (Table 2, maximum gas concentrations). Figure 3 illustrates this trend, showing the real-time monitoring of the CO concentration during the first 60 min after casting. Green sand’s elevated CO levels are attributed to the coal content in its composition, while organic binders release CO through the combustion of organic compounds (furfuryl alcohol in furan systems and phenolic compounds in alkaline phenolic systems being the primary contributors). In contrast, inorganic systems maintained CO levels below 3500 ppm, outperforming green systems by >30%. In combined systems using green sand molds with inorganic cores, no significant differences were observed compared to traditional green sand molds with phenolic urethane cores.

#### 2.5.2. NO_x_ Emission Profile Analysis

**Figure 4:**
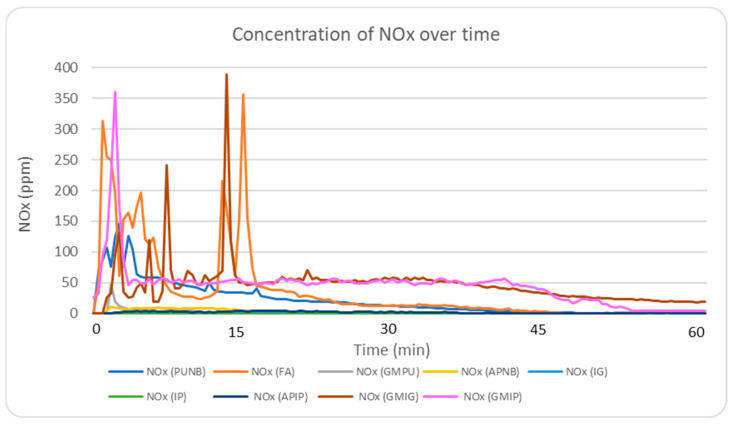
Real-time monitoring of NO_x_ concentrations during the experimental period for the nine different systems.

NO_x_ emissions were significantly reduced using inorganic binders, with levels falling below 2 ppm. In comparison, NO_x_ concentrations exceeded 350 ppm in green sand and FA systems, and were around 150 ppm in the PUNB system. This finding represents a reduction of more than 98% in NO_x_ emissions when using inorganic binders. Figure 4 shows real-time NO_x_ emission profiles during the first 60 min after casting. The presence of coal dust in green sand can lead to an increase in temperatures during the pouring process, promoting the formation of NO_x_ from nitrogen present in the air. Similarly, in the FA and PUNB systems, the exothermic reactions occurring during pouring contribute to the generation of NO_x_. Phenolic–alkaline systems released significantly less NO_x_ (5–11 ppm), though still higher than inorganic binders (1–2 ppm).

#### 2.5.3. SO_2_ Emission Profile Analysis

**Figure 5:**
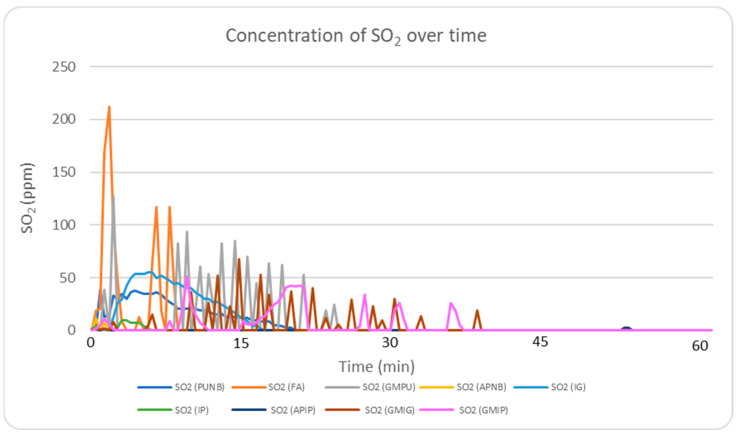
Real-time monitoring of SO_2_ concentrations during the experimental period for the nine different systems.

Figure 5 presents the real-time monitoring of SO_2_ concentrations during the first 60 min following casting. Among all binder systems, FA displayed the highest levels, reaching 212 ppm. This elevated concentration is attributed to the presence of sulfur compounds in its hardener (para-toluenesulfonic acid). Green sand systems showed the next highest SO_2_ emissions, with values exceeding 50 ppm, likely due to the carbon content in their composition. When comparing the FA system to the inorganic systems, inorganic binders showed a significant reduction of over 75% in SO_2_ emissions.

#### 2.5.4. O_2_ Consumption Profile Analysis

**Figure 6:**
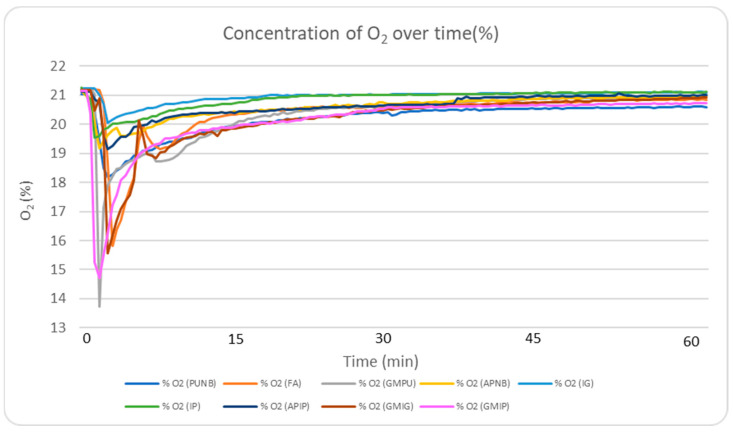
Real-time monitoring of O_2_ concentrations during the experimental period for the nine different systems.

In terms of oxygen consumption, the green sand, FA, and PUNB binder systems required more oxygen than the alkaline-phenolic and inorganic systems (Table 2, maximum gas concentrations). Figure 6 illustrates this trend, showing the O_2_ concentration profile during the first 60 min after casting. The data suggest an inverse relationship between oxygen consumption and the production of harmful combustion gases: as available oxygen is depleted, higher amounts of CO, NO_x_, and SO_2_ and harmful substances are generated. Inorganic binders demonstrated lower oxygen consumption, resulting in reduced gas generation and potentially minimizing the formation of surface pores on cast pieces.

#### 2.5.5. Temperature Profile Analysis

Temperature measurements taken at the sampling points of the gas duct, approximately 5 m from the pouring area, revealed significant variations across different binder systems. As outlined in Table 2, molds produced with FA, PUNB, and green sand systems reached higher temperature ranges (50–82 °C), whereas molds manufactured with alkaline–phenolic binders (34–41 °C) and inorganic binder systems (41–46 °C) showed lower temperatures. This phenomenon can be attributed to the rapid reaction of organic solvent-based systems and the carbon present in green sand with the elevated temperature of molten iron. These interactions lead to the decomposition of organic components, resulting in a more exothermic reaction. In contrast, water-based systems—such as alkaline phenolic and inorganic binders—undergo reactions that generate significantly less heat.

## 3. Materials and Methods

This study employed a comprehensive approach to investigate the environmental impacts of various binder systems in sand casting. The experimental setup was designed to simulate industrial casting conditions while allowing for precise measurement of emissions.

### 3.1. Sand and Binder System

The foundry sand used in this study was a high-quality silica sand with an AFS fineness number of 55.

Binder systems commonly used in the market, and which are representative of consortium foundries, were selected for the experiments. These binders included no-bake organic resins, no-bake inorganic binders, and bentonite-based green sand. The organic and inorganic binder systems were applied at concentrations ranging from 1% to 2.5% by weight of sand, as per supplier recommendations (Table 3). For the green sand system, a standard composition of 9% bentonite, 3% coal dust, and approximately 4% water was utilized, reflecting common industrial practice. The main components and concentration ranges of the different binders, based on supplier safety data sheets, are detailed in Table 4.

Considering these recipes, nine combinations of mold/core systems were tested, represented by the following codes:

- PUNB—Mold and core with Phenolic Urethane No-Bake binder
- FA—Furan (based on Furfuryl Alcohol)
- GMPU—Green mold (clay-bonded) + Phenolic Urethane No-Bake core
- APNB—Mold and core with Alkaline–Phenolic No-Bake
- IG—Mold and core with inorganic geopolymer
- IP—Mold and core with inorganic sodium silicate
- APIP—Alkaline–Phenolic mold+ Inorganic silicate core
- GMIG—Green mold (clay-bonded) + Inorganic geopolymer core
- GMIP—Green mold (clay-bonded) + Inorganic sodium silicate core

The mold and core combinations were carefully selected, taking into account both pH levels and the compatibility of materials used in molds and cores. This approach was adopted with the specific goal of future implementation in foundry operations.

### 3.2. Equipment Specifications

Molds and cores were prepared using a semi-industrial LORAMENDI X-100 mixer with a 50 kg capacity. This equipment ensured homogeneous mixing of sand and binder components, critical for consistent mold properties. The no-bake process was employed for all test combinations, without the use of protective coatings, in order to isolate the effects of the binder systems.

A custom-designed cylindrical pattern and core box were used to produce standardized molds. Each semi-mold contained approximately 50 kg of the prepared sand mixture, with cores weighing approximately 5 kg. This configuration resulted in cast parts weighing around 55 kg, providing a realistic representation of medium-sized industrial castings. The sand-to-metal ratio was 2:1.

For iron melting, a 100 kg capacity medium-frequency induction furnace (INDUCTOTHERM) was used, providing sufficient molten metal to conduct the experiments while ensuring consistent pouring temperatures.

### 3.3. Methodology and Test Setup

Emissions (BTEX, PAHs, phenol, crystalline silica, particulate matter, and combustion gases) were measured in closed chamber tests by pouring approximately 60 kg of molten iron into each 110 kg sand mold. When the molten metal is introduced into the sand molds, the thermal energy initiates the degradation, pyrolysis, and combustion of organic chemical binders and coal present in the mold and cores. This process leads to the formation and release of a diverse array of VOCs and Hazardous Air Pollutants (HAPs) as a result of incomplete combustion. Simultaneously, the mechanical disturbance of sand particles during pouring leads to the generation of significant amounts of particulate matter and dust emissions. These emissions include both filterable and condensable particulates, which pose environmental and health risks due to the potential presence of harmful substances such as metal oxides and silica.

**Figure 7:**
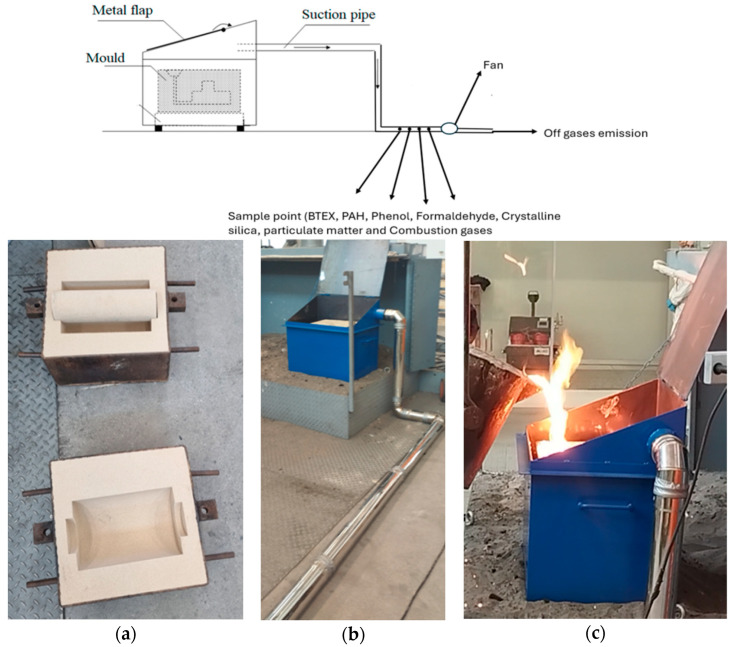
Experimental setup for measurements of emissions from sand castings. Figure adapted from [12] doi.org/10.3390/ma14102581

As illustrated in Figure 7, the use of a custom-designed metal enclosure with a closable top lid provides a controlled environment for emission testing. This setup allows for

- A controlled testing environment: The casting process is isolated from external variables, enabling more accurate comparisons between different molding systems;
- Efficient exhaust gas collection: A special connector and piping system with sampling holes ensure the capture of a wide range of emissions, offering a comprehensive and accurate view of their environmental impact.

### 3.4. Emission Measurement Techniques

The emission measurement techniques described in this section demonstrate a comprehensive approach for analyzing various hazardous pollutants emitted during the metal casting process. This methodology employs a range of sampling and analytical techniques to capture and quantify different types of emissions.

#### 3.4.1. Gas Sample Collection for BTEX, PAHs, and Phenol

Two specialized sampling pumps from SKC Inc. (Eighty-Four, PA, USA) were employed to collect various organic compounds. For BTEX and phenol sampling, the Pocket Pump TOUCH, with a flow range of 20–500 mL/min, was used in conjunction with sorbent tubes.

PAHs were collected using the Universal PCXR8 Sample Pump, which features a flow range of 50–5000 mL/min, coupled with filters and sorbent tubes.

This comprehensive setup enabled flexible and precise sampling of a wide spectrum of organic compounds in both gas and vapor forms, ensuring accurate data collection across different chemical groups.

After sampling, the extracted gases were analyzed using gas chromatography with flame ionization detection (GC-FID) for BTEX compounds (PLE-16, based on UNE 81586:2020 [14]) and high-performance liquid chromatography with fluorescence detection (HPLC-FLD) for PAHs (ITA-CL-061, based on NIOSH 5506 Standard [15]). This approach enabled comprehensive chemical characterization.

#### 3.4.2. Real-Time Combustion Gas Analysis

A TESTO 350 flue gas analyzer (Testo SE & Co. KGaA, Lenzkirch, Germany) was used for the real-time measurement of exhaust gases (CO, O_2_, NO_x_, and SO_2_) during pouring and cooldown processes. This setup allowed for a time-dependent analysis of the fume composition. Electrochemical cells were used for detecting CO, O_2_, NO_x_, and SO_2_ concentrations with high sensitivity. This advanced analytical approach offers several advantages, including precise multi-gas detection and real-time data acquisition for emission monitoring, and the ability to track temporal variations in emissions.

#### 3.4.3. Dust and Crystalline Silica Sampling

Universal PCXR8 Sample Pumps (SKC Inc., Eighty Four, PA, USA), featuring a flow range of 50–5000 mL/min, were used for particulate matter and crystalline silica sampling, in accordance with the following standards:

- Crystalline silica: 37 mm PVC filter cassette with Higgins & Dewell cyclone; analyzed by infrared spectroscopy (UNE 81550:2017 standard [16]);
- Particulate matter: Pre-weighed 37 mm PVC filter with Higgins & Dewell cyclone; analyzed by gravimetry (UNE 81599:2014 standard [17]).

This approach allows for the quantification of respirable dust and specific analysis of crystalline silica content, which is crucial for assessing occupational health risks in foundry environments, given the increasingly stringent limit values (0.05 mg/m^3^).

The combination of these techniques provides a comprehensive emissions profile for the casting process, enabling a thorough evaluation of the environmental and health impacts of different binder systems. These data are essential for comparing the performance of inorganic binders against traditional organic binders in terms of the reduction of emissions and overall environmental footprint.

## 4. Conclusions and Future Perspectives

The tests were conducted in a controlled chamber setup, minimizing external factors and enabling a direct comparison of different molding/core systems to more accurately assess their environmental impacts.

The elevated temperatures during casting production cause thermal decomposition of organic components in the molding sand, leading to the release of harmful gaseous and volatile compounds (BTEX, PAH, phenol, CO, NO_x_, SO_2_), as well as particulate substances.

From the nine measuring tests conducted, the following conclusions can be drawn:

- The data show that emissions are significantly higher for molds and cores produced using organic binders, such as FA, PUNB, and AP, compared to those manufactured using inorganic binder systems (IG and IP).
- The use of inorganic binders led to a significant reduction in BTEX emissions, with decreases greater than 90% compared to organic binder systems and over 65% compared to green molding systems. For all systems investigated except the furan-based one, benzene was the largest contributor to total BTEX emissions.
- For PAH emissions, inorganic binders achieved reductions of over 94% compared to organic binders and more than 90% compared to green molding sands.
- Concerning particulate matter, the highest emissions corresponded to sand bonded with FA and PUNB systems, followed by GMPU. However, these values carry significant uncertainty, as factors such as the method of pouring molten iron into the mold can influence particulate emissions.
- Green sand systems exhibited the highest CO emissions, attributed to their coal content, followed by organic binders. Inorganic systems showed significantly lower emissions, outperforming green sand by over 30%. Combined systems did not show notable differences compared to traditional setups.
- NO_x_ emissions were reduced by more than 98% when using inorganic binders, compared to green sand, FA, and PUNB.
- FA systems exhibited the highest SO_2_ emissions, likely due to the presence of sulfur compounds in their hardener. In contrast, inorganic binders achieved reductions of over 75%.
- Green sand, FA, and PUNB systems consumed more oxygen than alkaline–phenolic and inorganic binder systems. As oxygen availability decreases, higher amounts of CO, NO_x_, SO_2_, and other harmful substances are generated.
- The higher temperatures in organic solvent-based systems and green sand are caused by rapid reactions with molten iron, leading to more exothermic responses. In contrast, water-based systems such as alkaline–phenolic and inorganic binders generate less heat due to milder reactions.

These results collectively indicate that inorganic binder systems significantly outperform organic binders and green sand in reducing gas emissions during casting processes. Their low emission profiles make them an environmentally friendly alternative, aligning with sustainable manufacturing goals. Furthermore, the reductions in gas emissions are anticipated to positively impact the occurrence of casting defects such as gas porosity and surface pores, potentially improving the overall quality of cast components. However, due to the inorganic nature of these binder systems, there are still technical challenges to be addressed, particularly related to the collapsibility of complex cores, shake-out processes, and the reclamation of inorganic binders. These issues will be addressed in future work, carried out as part of the Greencasting LIFE project.

## Figures and Tables

**Table 1:** Test codes of the nine combinations of mold/core systems.

Test Code	Mold Binder	Core Binder
PUNB	Phenolic Urethane No-Bake	Phenolic Urethane No-Bake
FA	Furan	Furan
GMPU	Green (clay-bonded)	Phenolic Urethane No-Bake
APNB	Alkaline Phenolic No-Bake	Alkaline Phenolic No-Bake
IG	Inorganic geopolymer	Inorganic geopolymer
IP	Inorganic sodium silicate	Inorganic sodium silicate
APIP	Alkaline Phenolic No-Bake	Inorganic sodium silicate
GMIG	Green (clay-bonded)	Inorganic geopolymer
GMIP	Green (clay-bonded)	Inorganic sodium silicate

**Table 2:** Detailed analysis of exhaust gases from nine combinations of mold/core systems. Data include BTEX, PAH, phenol, particulate matter, and gases.

	Pyrolysis Products	PUNB (mg/kg)	FA (mg/kg)	GMPU (mg/kg)	APNB (mg/kg)	IG (mg/kg)	IP (mg/kg)	APIP (mg/kg)	GMIG (mg/kg)	GMIP (mg/kg)
**BTEX**	Benzene *	159.1	100.2	18.5	56.1	7.7	4.4	102.4	17.9	14.6
	Toluene	17.7	207.5	4.5	8	1.6	<0.7	13	7.3	6.8
	Ethylbenzene **	<0.9	1.6	<0.7	<0.8	<0.7	<0.7	<0.7	<0.7	<0.7
	Xylenes	2.3	14.4	1.5	2.8	<2.2	<2.2	4.7	3.6	2.8
	**Σ BTEX**	**179.5**	**323.7**	**24.8**	**67.3**	**10.8**	**6.3**	**120.5**	**29.1**	**24.6**
**PAH**	Acenaphthene + Fluorene	0.67	<0.77	0.7	2.83	0.13	<0.020	<0.08	(a)	1.42
	Acenaphthylene	0.96	<0.51	1.18	<0.30	<0.014	<0.014	2.03	(a)	<0.01
	Anthracene	0.33	0.76	0.034	0.02	<0.0007	<0.0007	0.11	(a)	0.005
	Benzo[a]anthracene	0.23	0.51	0.136	0.19	0.017	<0.0003	0.06	(a)	0.03
	Benzo(a)pyrene *	0.44	0.034	0.077	0.057	0.082	<0.0003	0.02	(a)	0.02
	Benzo[b]fluoranthene **	0.45	0.16	0.058	0.234	0.01	<0.0003	0.08	(a)	0.01
	Benzo[g,h,i]perylene *	<0.004	<0.015	0.048	0.075	0.007	<0.0003	0.04	(a)	0.01
	Benzo[k]fluoranthene **	0.04	0.054	0.039	0.234	0.004	<0.0003	0.03	(a)	0.002
	Chrysene	<0.004	<0.015	0.097	0.009	0.013	<0.0003	0.09	(a)	0.08
	Dibenz[a,h]anthracene **	0.095	0.018	0.049	0.118	0.015	<0.0003	0.05	(a)	<0.0004
	Phenanthrene	0.031	<0.026	0.046	<0.0151	<0.001	<0.0007	0.11	(a)	0.04
	Fluoranthene	1.43	0.721	0.097	0.093	0.011	<0.0003	0.03	(a)	0.02
	Indene (1, 2, 3-cd) pyrene **	<0.024	0.084	<0.002	<0.060	<0.003	<0.0027	0.03	(a)	0.01
	Naphthalene	0.445	2.66	<0.005	0.121	<0.006	<0.0054	1.55	(a)	1.07
	Pyrene	0.493	0.22	0.06	0.049	0.01	<0.0003	0.02	(a)	0.01
	**Σ PAHs**	**5.63**	**5.86**	**2.63**	**4.18**	**0.25**	**<0.046**	**4.20**	(a)	**2.73**
**Phenols**	Phenol	<4.5	-	<1.8	<4.2	-	-	<2.2	-	-
**Particulatte** **Matter**	Crystaline silica * (mg/kg)	<0.07	0.85	<0.13	<0.25	<0.03	<0.03	<0.07	<0.07	<0.03
	Particulate matter (mg/kg)	80.4	90.9	76.7	15	3.6	<0.67	3.9	2.5	7.2
**Gases**	CO max value (ppm)	302	**>5000**	**>5000**	4335	3467	3291	4456	**>5000**	**>5000**
	NO_x_ max value (ppm)	**147**	**356**	48	11	2	1	5	**388**	**360**
	SO_2_ max value (ppm)	39	**212**	**127**	11	55	10	14	68	51
	O_2_ max value (%)	18.1	**15.8**	**13.7**	19.2	20.1	19.6	19.2	**15.6**	**14.7**
	T° (max) in sampling points	56.7 °C	82 °C	60.5 °C	34.1 °C	39.5 °C	46.2 °C	41.2 °C	63.8 °C	50.3 °C
	Minutes to achieve T° max (min)	9	3	4	10	107	170	5	3	3

* Considered human carcinogenic by the U.S. Environmental Protection Agency (EPA), the European Union, and/or the International Agency for Research on Cancer (IARC); ** considered probable or possible human carcinogenic by the U.S. Environmental Protection Agency (EPA), the European Union, and/or the International Agency for Research on Cancer (IARC); the “<” sign means that the test result was obtained below the limit of the accreditation scope, which is not the result but only the information about the level of content/concentration of the tested factor. The numerical value after the < sign is the lower limit of the measurement range of the accredited method. (a) No data available for GMIG due to contamination during handling in the laboratory analysis.

**Table 3:** Dosage of each binder system according to the supplier.

System	Binder (%) Over Sand	Hardener (%) Over Binder
Phenolic Urethane No-Bake (PUNB)	1.2 (R1 + R2)	5 (over R1)
Furan (FU)	1	30
Alkaline-Phenolic No-Bake (APNB)	1.7	20
Geopolymer (IG)	2	16
Sodium silicate (IP)	2.5	10
Green (GM)	9% bentonite/3% coal dust/4% water	

**Table 4:** Binder systems evaluated in this study and their approximate formulation according to safety data sheets.

Binder	Binder Type	Composition According to Supplier [wt%]	
PUNB	Part I	Phenol	3–5
		Formaldehyde	0.00–0.1
	Part II	Diphenylmethane-4-4′-diisocyanate	75–100
		Solvent naphtha	10–20
		Carbohydrates C11-C14, N-Alkanes, Isoalkanes, Cyclics, with less than 2% Aromatic Compounds	5–10
		Polymethylene Polyphenyl Isocyanate	0.00–0.2
	Catalyst	4-(3-Phenylpropyl)pyridine	23–75
FA	Binder	Furfuryl alcohol	30–75 (65%)
		Free Phenol	0.6–0.8
		Free Formaldehyde	0.00–0.1
	Catalyst	Para-Toluenesulfonic acid	65–70
APNB	Binder	Phenol, a polymer with formaldehyde	35–50
		Sodium hydroxide.	5–10
		Potassium hydroxide	2–3
	Catalyst	Gamma-butyrolactone	<20
		Propylene carbonate	<20
IG	Binder	Silicic acid, sodium salt	35–40%
		Aluminum sodium oxide	<5%
	Catalyst	Diethyleglykol	5–20%
IP	Binder	Silicic acid, sodium salt	25–50
		Disodium octaborate tetrahydrate	<2
		Ethanediol	<1
	Catalyst	Propylene carbonate	10–40

## Data Availability

The original contributions presented in the study are included in the article; further inquiries can be directed to the corresponding authors.

## Abbreviations

The following abbreviations are used in this manuscript:

VOCs	Volatile Organic Compounds
PAHs	Polycyclic Aromatic Hydrocarbons
BTEX	Benzene, Toluene, Ethylbenzene, and Xylene
PUNB	Phenolic Urethane No-Bake
FA	Furan (based on Furfuryl Alcohol)
APNB	Alkaline–Phenolic No-Bake
IG	Inorganic Geopolymer
IP	Inorganic Sodium Silicate
GM	Green Mold
HAPs	Hazardous Air Pollutants
GC-FID	Gas Chromatography with Flame Ionization Detection
HPLC-FLD	High-Performance Liquid Chromatography with Fluorescence Detection
